# Genetic diversity and population structure of the endangered orchid *Pelatantheria scolopendrifolia* (Orchidaceae) in Korea

**DOI:** 10.1371/journal.pone.0237546

**Published:** 2020-08-13

**Authors:** Seon A. Yun, Hyun-Deok Son, Hyoung-Tak Im, Seung-Chul Kim

**Affiliations:** 1 Department of Biological Sciences, Sungkyunkwan University, Suwon, Gyeonggi-do, Korea; 2 Ministry of Environment, Sejong, Korea; 3 Department of Biological Sciences, Chonnam National University, Gwangju, Korea; USDA-ARS Southern Regional Research Center, UNITED STATES

## Abstract

Due to substantial population decline, the Korean orchid *P*. *scolopendrifolia* is considered endangered and highly threatened. Like many endangered species, it is vulnerable to biological and anthropogenic threats that can lead to the loss of genetic diversity and, ultimately, extinction. Therefore, the assessment of genetic diversity and population genetic structure is imperative for conservation. In this study, we newly developed 15 polymorphic microsatellite markers. Analyses of genetic diversity and population genetic structure that included 182 samples from 11 populations were conducted using microsatellite markers and four noncoding regions of chloroplast DNA. Our study revealed a relatively low level of genetic diversity (*Ho* = 0.529, *He* = 0.356), albeit harboring with private alleles based on microsatellite genotyping data, and high haplotype diversities based on chloroplast DNA sequences data. The results of STRUCTURE and PCoA based on microsatellite genotyping data showed population differentiations. An AMOVA based on chloroplast DNA sequence data further corroborated these conclusions, indicating about 70% of variations found among populations. Low genetic diversity and divergence among the population might have been caused by factors, such as asexual reproduction, demographic events (bottleneck and population expansion), geographic isolation, and low gene flow. The development and implication of conservation strategies and management of *P*. *scolopendrifolia* are proposed based on these results.

## Introduction

Species with small population size or small geographic ranges subsequently tend to have reduced gene flow and increased genetic divergence, due to genetic drift and inbreeding [1]. In particular, many endangered species have low genetic diversity and undergone population differentiation as a result of overexploitation [2, 3] and habitat disturbance [4–6]. Because vulnerabilities to biological and anthropogenic threats are directly linked to loss of genetic diversity and extinction, the assessment of genetic diversity and population genetic structure is imperative for development and implication of conservation strategies.

The family Orchidaceae is one of the most species-rich families, and this high diversity along with species-specific pollination has attracted numerous biologists [7–9]. Pollination success may affect the long-term survival of population or species, but it strongly depends on the presence of pollinator. Therefore, it may be vulnerable to anthropogenic threats, such as habitat disturbance, which affect both pollinator and orchids. The overexploitation of natural populations by gathering for ornamental and medicinal uses has been a major subject of conservation issues, and at least recently, awareness about the conservation of biodiversity has grown. In addition, terrestrial orchids are known to have small seeds, and germination is difficult due to requirements of specific nutrients and environmental conditions [10–12]. These factors may make restoration and conservation of plant at risk difficult and challenging. Given the conservation priority of highly threatened orchid species, several studies have been conducted, evaluating genetic diversity and structure based on various molecular markers, such as isozymes [13], allozymes [14, 15], AFLPs [amplified fragment length polymorphisms; [16–18]], ISSRs [inter simple sequence repeats; [19]], and SSRs [simple sequence repeats or microsatellites; [20, 21]]. In particular, studies of microsatellite markers remain popular in population genetics and conservation genetics because these markers are co-dominant, highly polymorphic, selectively neutral, and have high mutation rates [22, 23]. Chloroplast (cp) DNA markers are also useful for evaluating population genetic diversity and structure [24–26] as well as phylogenetic analyses [27–29] because of non-recombinant and uniparental inheritance [30].

*Pelatantheria scolopendrifolia* (Makino) Aver. [= *Cleisostoma scolopendrifolium* (Makino) Garay] is an epiphytic or lithophytic evergreen perennial orchid, belonging to subtribe Aeridinae (Orchidaceae), and occurs across Korea, China, and Japan. In Korea, *P*. *scolopendrifolia* is highly restricted to southwestern corner of the Korean Peninsula, including coastal (i.e., Mokpo and Haenam) and inland (i.e., Naju) regions as well as islands of the southwest (i.e., Jeju, Bogil, Jindo, and Dongrae). With a narrow extent of occurrence (EOO, 2,000 km^2^) and substantial population decline due to anthropogenic activities, such as illegal collection and habitat disturbances, the species is assessed as EN B2b(iii,iv)c(iii,iv,v) on Korean Red list and protected by law as Endangered Wildlife [31]. The rarity of this species and its need for conservation have influenced studies of *P*. *scolopendrifolia*. For example, given the fact that genetic diversity is particularly important to the conservation of rare or endemic species [32], allozyme-based and microsatellite-based studies were carried out, providing some baseline genetic information for conservation [2, 33]. Their studies, however, yielded somewhat conflicting results; low genetic diversity within populations (*He* = 0.002) based on allozymes [2] versus relatively high genetic diversity (*He* = 0.4162) based on microsatellite markers [33]. In addition to population genetic studies, Son et al. [34] reported the male megachilid bee (*Megachile yasumatsui*) as a pollinator and self-incompatibility of this species. Furthermore, Son et al. [35] also presented a positive relationship between the frequencies of pollinator visits and fruiting rates. Given that preliminary studies have shown contradictory results, further genetic studies of *P*. *scolopendrifolia* using various markers and extensive sampling are necessary to provide more comprehensive genetic information and also to develop conservation guidances based on this genetic perspective.

Thus, the present study aimed to develop additional microsatellite markers of *P*. *scolopendrifolia* and to determine the genetic diversity, population genetic structure, and the factors that contribute to genetic patterns using the newly developed markers and four non-coding chloroplast DNA regions. Based on the findings of this study, we proposed several baseline guidelines or suggestions for conservation and management strategies of highly threatened species of *P*. *scolopendrifolia* in Korea.

## Materials and methods

### Plant materials

We sampled *P*. *scolopendrifolia* from 11 populations, including two in Japan, to analyze genetic diversity and structure (Table 1). In Korea, we collected a total of 166 individuals, including inland population from Naju (NJ; 32 individuals), two coastal populations from Mokpo (MP-1, 5 individuals and MP-2, 13 individuals), and one in-land (HN-1; 14 individuals) and one coastal (HN-2; 7 individuals) population from Haenam region. Samples were also collected from the island of Jindo (JD-1, 58 individuals and JD-2, 11 individuals), Gwanmaedo (GM; 6 individuals), and Wando (WD; 20 individuals). Lastly, a total of 16 individuals from two populations (eight individuals per population) in Japan were also included as island populations to understand the origin of *P*. *scolopendrifolia* in Korea. These have been transplanted from natural population in Kyushu to the Batanic Garden for conservation. All necessary permits were obtained for the described study, which complied with all relevant regulations. The number of individuals randomly sampled per population was proportional to population size (raning from five in small populations to 58 in large populations), and a minimum collecting distance of 20 cm between sampled individuals was used to prevent resampling the same individual. All leaf samples were dried with silica gel, and voucher specimens for each population were deposited in the Chonnam National University Herbarium (CNU).

**Table 1:** Sampling information and number of haplotypes found in each population.

Region	Population code	Sample size	Locality	No. of Haplotype	Haplotypes (number of individuals)
Korea					
Naju (NJ)	NJ	32	Dado-myeon, Naju-si, Jeollanam-do	3	J (30), K (1), M (1)
Mokpo (MP)	MP-1	5	Haeyangdaehak-ro, Mokpo-si, Jeollanam-do	2	O (4), P (1)
	MP-2	13	Udal-ro, Mokpo-si, Jeollanam-do	1	O (13)
Haenam (HN)	HN-1	14	Haenam-eup, Haenam-gun, Jeollanam-do	1	O (14)
	HN-2	7	Songji-myeon, Haenam-gun, Jeollanam-do	2	A (6), F (1)
Jindo (JD)	JD-1	58	Gunnae-myeon, Jindo-gun, Jeollanam-do	10	A (34), B (1), C (1), D (2), E (5), L (2), O (5), Q (1), R (6), S (1)
	JD-2	11	Uisin-myeon, Jindo-gun, Jeollanam-do	7	A (1), H (2), I (1), J (3), M (1), O (2), T (1)
Gwanmaedo (GM)	GM	6	Jodo-myeon, Jindo-gun, Jeollanam-do	3	H (2), J (3), M (1)
Wando (WD)	WD	20	Wando-eup, Wando-gun, Jeollanam-do	2	A (18), E (2)
Japan					
	JPN-1	8	Godaisan, Kochi-si, Kochi-hyeon	2	G (2), N (6)
	JPN-2	8	Godaisan, Kochi-si, Kochi-hyeon	1	O (8)
Total		182			

The coordinates in latitude/longitude of the collection site are not provided for the protection.

### DNA extraction and microsatellite markers development

Total DNA was extracted using the DNeasy Plant Mini Kit (Qiagen, Carlsbad, California, USA), following the instructions of the manufacturer. The leaves sampled from two representative populations, coastal population Mokpo (MP-2, with observed pollinators) and inland population Naju (NJ, without observed pollinators) were specifically selected to develop the microsatellite markers of *P*. *scolopendrifolia*. According to the MiSeq platform, an Illumina paired-end genomic library was constructed using the TruSeq DNA LT Sample Prep Kit (Illumina, San Diego, California, USA) and sequenced 11,173,080 and 8,146,500 paired-end reads from MP-2 and NJ individuals, respectively. Each paired-end read was 301 base pairs (bp) in length after trimming. The adaptor sequences and low-quality reads (< 36 bp) were trimmed by Scythe v0.994 and Sickle v1.33, respectively. The trimmed sequencing data were registered at NCBI Sequence Read Archive (SRA BioProject accessions MP-2: PRJNA565547 and NJ: PRJNA625297) and pair-end reads were merged into one read using the BBMerge program in Geneious ver. 10.2.2 [36]. The PrimerPipeline program which incorporates MIcroSAtellite identification software (MISA) [37] and Primer3 [38] was then used for mining the microsatellites. In order to search SSR loci, the following minimum thresholds were applied: di-, tri-, tetranucleotide with eight repeats, pentanucleotide with seven repeats, and hexanucleotide with six repeats. The primer set was designed according to the following parameters: annealing temperature of 57–63°C, product size of 100-300bp, and length of 18-27bp. By comparing the results obtained from MP-2 and NJ individuals, 32 primer pairs with the same motif and different repeat number were preferentially selected, and then 22 primer pairs were randomly chosen from the SSR candidate list of MP-2 individual. Then, 54 primer pairs labeled with either 6-FAM or HEX fluorescent dye were used for initial polymorphism tests for eight individuals from six population (NJ, MP-1, MP-2, WD, HN-1, and JD-1). The 15 primers pairs which had confirmed polymorphism were finally applied to genotype the remaining 174 individuals.

PCR amplifications were performed in a total volume of 15μL containing 0.5μL of genomic template DNA, 0.2μL of 10 pmol forward primer with fluorescence dye, 0.2μL of 10 pmol reverse primer, 1.0μL (with 2.5 mM MgCl_2_) of PCR buffer, 0.2μL (each 10 mM) of dNTPs, and 0.05μL (5 U/μL) of Taq DNA polymerase (Inclone Biotech, Gyeonggi-do, Korea). PCR conditions were an initial denaturation at 95°C for 3 min; followed by 35 cycles at 95°C for 1 min, annealing at 60°C for 1 min, extension at 72°C for 2 min, and a final extension at 72°C for 5 min. PCR amplicons were then separated with a GeneScan 500 LIZ Size Standard (Applied Biosystems, USA) at the Macrogen Company (Seoul, Korea) utilizing ABI 3730XL DNA Sequencer (Applied Biosystems, USA). Allele size was scored using GeneMapper v5.0 (Applied Biosystems).

### Microsatellite genotyping and chloroplast DNA sequencing

To analyze the genetic diversity and population structure of *P*. *scolopendrifolia*, 15 newly developed microsatellite markers in this study and additional three microsatellite markers developed by Han [33] were used (Table 2). PCR amplification and detection of allele size were conducted in the same conditions as described previously.

**Table 2:** List of 21 microsatellite markers used in this study.

Locus	Primer sequences (5’-3’)	Repeat motif	Annealing Ta (°C)	Expected size (bp)	Allele size range (bp)	GenBank accession no.
**PS02**	F: AGGTGCTCTTTACCTTGAGGA	(CT)_14_	60	170	168–174	MN592878
	R: TGGACCCACCATGCATAACA					
**PS06**	F: ACAACAACGCTTCCTTTGGC	(AG)_18_	60	161	155–171	MN592879
	R: CCCAAACCTCTCCTATCGCA					
PS21*	F: GCGCCAAAGGATCCATATATGC	(GT)_12_	60	255	255	MN592880
	R: TTGCATCGATGGGATTGCAT					
**PS24**	F: ACATGAGTGATAGAAGATGCACCA	(ACA)_6_	60	202	199–256	MN592881
	R: GACCTAGGAAGACTTGGGCG					
**PS25**	F: ATGAGTGCTGCTGTGATCCG	(GA)_11_	60	200	194–210	MN592882
	R: TCATCTCACCTTAAAGCATGCA					
**PS29**	F: TGCAGATGAAAACAAGTCTTCCA	(AT)_10_	60	155	143–145	MN592883
	R: TAAGCAGGGACACACTGCTG					
PS30*	F: TCTATAGACAATCTGCTTCCCCA	(CAA)_9_	60	199	199	MN592884
	R: AAGGCAGGGGTAGACCTAGG					
**PS31**	F: GATATTCCCTCCTAGCGCGG	(CT)_12_	60	252	220–260	MN592885
	R: GACCTTATAATGGCTCCACCCA					
**PS32**	F: AGCACCACCTAGACTAACTTGA	(TC)_12_	60	232	232–254	MN592886
	R: TGGTATGTAAGGGAATACCATGTCT					
**PS36**	F: ACGTTAACAAAGCTCACAACAGT	(TCT)_19_	60	272	263–284	MN592887
	R: AGCCCAGAACCTAGCAATGA					
**PS37**	F: AGCTAAGTTGCACGGCCTAA	(TACA)_9_	60	196	196–200	MN592888
	R: TCTGGTATGAAAGATATGGGTGGT					
**PS40**	F: TCCATTGCCACCACCTACAC	(CCT)_10_	60	268	265–274	MN592889
	R: GCACATCATCAACGTCGCTC					
**PS43**	F: CGTCACTCAGGATCAATCTGGT	(AAC)_17_	60	224	200–224	MN592890
	R: GACCTAGGAAGACTTGGGCG					
**PS44**	F: AGCTTCACTCGTTACAAGGCA	(AAG)_9_(ATG)_9_	60	171	144–171	MN592891
	R: ACACAACAAGTTCCAATCCACA					
**PS47**	F: ACGATGAGTGAAGGAGAAAAGGA	(ATC)_8_	60	279	270–279	MN592892
	R: TGCTACCATGTGCATCTCAGT					
**PS51**	F: AGGTGTTGACTGCACATATAGCT	(TCA)_10_	60	169	148–169	MN592893
	R: TGGGCTAATGGCATTGATGT					
**PS52**	F: ACGGTAGTCCTCAGTGCAAC	(TGA)_17_	60	224	179–197	MN592894
	R: ACCTAAGCTCGCTCGAACTC					
PS54*	F: AGAAAGAGGAGTCAGGTTCGT	(TTTTCT)_6_	60	268	268	MN592895
	R: TCGAGGCCGAAGTAGAGGAC					
**CW3164**	F: ACAAACATCGAGCAGAAAGGC	(GAA)_23_	60	169	130–166	-
	R: AGGTATAAAGGGGGCAGAGC					
**CW3189**	F: TCGTAAAGTCCATAGGTGGTCC	(ATA)_23_	60	163	145–163	-
	R: ACAACACTAATGAATGGTGCC					
**CW3248**	F: TCGTTGTCGTCGTCATTACC	(ATT)_21_	60	316	295–322	-
	R: TGCCCCGTTATGTATGAAAAGG					

In this study, we developed a total of 18 microsatellite markers, of which 15 are polymorphic, while the remaining three are monomorphic. All sequences were deposited at GenBank. Three markers (CW3164, CW3189, and CW3248) were previously developed in P. scolopendrifolia by Han [33]. Asterisk (*) indicates a monomorphic marker and exclusion from this study.

For the selection of cp DNA markers with variations within and between populations, 17 chloroplast non-coding or intergenic spacer regions (*acc*D- *psa*I, *atp*I-*atp*H, *pet*A-*psb*J, *pet*L-*psb*E, *psb*A-*trn*H, *psb*B-*psb*H, *rpl*14-*rpl*36, *rpl*16 intron, *rpl*32-*trn*L, *rpo*B-*trn*C, *rps*12-*rpl*20, *rps*16 intron, *trn*C-*ycf*6, *trn*K- *rps*16, *trn*L-*trn*F, *trn*S-*trn*G, and *ycf*6-*psb*M) [39] were tested using the same eight individuals used to develop the microsatellite markers. Although all but three regions were successfully amplified, four markers (*acc*D- *psa*I, *pet*L*-psb*E, *psb*A*-trn*H, and *rps*12*-rpl*20) with variations within and between populations were ultimately selected. PCR amplifications were performed in a total volume of 50μL containing 1.0μL of genomic template DNA, 1.0μL of 10 pmol forward primer, 1.0μL of 10 pmol reverse primer, 5.0μL (with 2.5mM MgCl_2_) of PCR buffer, 1.0μL (each 10mM) of dNTPs, and 0.25μL (5U/μL) of Taq DNA polymerase (Inclone Biotech, Gyeonggi do, Korea). The PCR conditions were initial denaturation at 95°C for 2 min, 35 cycles at 95°C for 1 min, annealing at 54°C for 1 min, extension at 72°C for 2 min, and a final extension at 72°C for 5 min. All PCR products were confirmed on 1% agarose gels and purified with Inclone^™^ Gel & PCR purification kit (Inclone Biotech, Gyeonggi do, Korea). Direct sequencing of PCR products was performed at Macrogen (Seoul, Korea). Sequence editing and alignment were conducted using Geneious 10.2.2 [36].

### Data analysis

Microchecker 2.2.3 [40] was used to detect evidence of null alleles and potential genotyping errors. Null allele frequencies for each locus were estimated with the expectation maximization (EM) algorithm described in Dempster et al. [41] using the program FreeNA [42]. Genetic diversity indices, such as the number of different alleles (*Na*), number of effective alleles (*Ne*), observed heterozygosity (*Ho*), expected heterozygosity (*He*), proportion of polymorphic loci (*P*), and private alleles (*Pa*), were estimated based on the microsatellite allele frequencies over 18 loci using GenAlEx v. 6.5 [43]. Allelic richness (*Ar*) was calculated based on minimum sample size of 5 individuals in each of the 11 populations using FSTAT 2.9.4. software [44]. Deviations from Hardy-Weinberg Equilibrium (HWE) and Linkage Disequilibrium (LD) were estimated using Genepop version 4.2 [45]. The genetic differentiation coefficient (*F*_*IS* and_ *Fst*) was estimated for all populations, and an analysis of molecular variance (AMOVA) was conducted with 999 permutations at two hierarchical levels (among populations and within populations) using GenAlEx v. 6.5 [43]. Principal coordinate analysis (PCoA) was carried out based on a codominant genetic distance matrix to examine genetic similarities and relationships between individuals and among populations using GenAlEx v. 6.5 [43]. A Mantel test [46] was conducted to determine correlations between genetic and geographic distance using GenAlEx v. 6.5 [43] with 999 permutations. For this analysis, we utilized the two datasets: one included all populations while the other consisted of nine Korean populations only. The genetic structure of populations was analyzed based on a Bayesian algorithm by STRUCTURE v.2.3.4 [47]. The admixture model was used with correlated allele frequencies applying burn-in of 2 x 10^5^ and runs of 2 x 10^5^ repetitions. Twenty runs were conducted for each value of potential genetic clusters (K), with K ranging from 1 to 15. The best K was identified based on the approach of Evanno et al. [48] using the program STRUCTURE HARVESTER v.0.6.94 [49]. The graphical results were displayed using CLUMPAK [50]. Bottleneck analysis was performed using BOTTLENECK v.1.2.02 software [51]. Heterozygosity excess caused by recent population bottlenecks was tested using a one-tailed Wilcoxon signed-rank test under a two-phase model (TPM) with a variance of 12% and a 70% proportion of a stepwise mutation model in the TPM. Mode-shift tests were also used to detect genetic bottlenecks.

The four combined cp DNA data were used to construct an unrooted haplotype network using the statistical parsimony approach [52] as implemented in TCS 1.21 [53]. The analysis was run with gaps coded as missing data, and the connection limit excluding homoplastic changes set to 95% following Hart and Sunday [54]. Furthermore, consecutive point-mutations were considered as a single-step mutation event, and messy poly(A)/(T) regions were deleted in this analysis. Genetic diversity parameters such as nucleotide diversity and gene diversity were calculated using Arlequin version 3.5 [55]. Selective neutrality indices, Tajima’s *D* [56] and Fu’s *Fs* test [57], were conducted to assess population expansion. In addition, an analysis of molecular variance (AMOVA) implemented using Arlequin version 3.5 [55] was employed to estimate the genetic variation among and within populations. Haplotype sequences of *P*. *scolopendrifolia* were deposited in GenBank: MT333568-MT333587 for *rpl*20-*rps*12, MT333588-MT333607 for *pet*A-*psb*J, MT333608-MT333627 for *acc*D-*psa*I, and MT333628-MT333647 for *pet*L-*psb*E.

## Results

### Development of novel polymorphic microsatellite markers

Among the 54 paired primers designed and synthesized, 15 polymorphic and 3 monomorphic microsatellite markers were newly developed for *P*. *scolopendrifolia* (Table 2). Repeat motifs were detected 6 dinucleotides, 7 trinucleotides, 1 tetranucleotides, and 1 complex. The allele size ranged from 143 to 284 bp and the number of alleles per locus ranged from 2 to 9, with an average of 5.13 alleles per locus. Eighteen newly developed microsatellite markers were deposited at GenBank (MN592878-MN592895) and 15 polymorphic markers were used in this study.

### Genetic diversity based on microsatellite markers

The missing data included in the final data set showed a low frequency of 0.15%. The presence of null alleles was checked using Microchecker 2.2.3 [40], and there were no regular patterns in the existence of null alleles in genotyping. While populations MP-1, MP-2, HN-1, GM, JPN-1, and JPN-2 detected no null alleles, the rest of the populations had null allele(s) at one or two loci; locus PS25 in HN-2, loci PS6 and CW3164 in JD-1, loci PS6 and CW3189 in JD-2, loci PS40 and PS43 in NJ, and loci PS25 and PS29 in WD (Table 4). The program FreeNa [42] was used to calculate null allele frequencies and created global *Fst* values across all loci. The *Fst* values (*Fst* = 0.29697, 95% CI (confidence interval) = 0.24707–0.34803) after excluding null alleles (ENA) were similar to the *Fst* values (*Fst* = 0.30185, 95% CI = 0.25362–0.35281) derived from raw microsatellite data, suggesting that the effect of null alleles on the genetic structure of populations is likely negligible. Twelve loci deviated significantly from Hardy-Weinberg equilibrium (Table 3). Tests for the significance of linkage disequilibrium over all loci and populations showed linkage disequilibrium between all but 33 pairs of loci (S1 Table).

**Table 3:** Genetic diversity parameters assessed for 18 SSR loci across 182 individuals from 11 populations of *P*. *scolopendrifolia*.

Locus	*Na*	*Ne*	*Ho*	*He*	*Fst*^*a*^	*Fst*^*b*^	*F*_*IS*_	HWE
PS02	1.364	1.204	0.158	0.119	0.472	0.476	-0.203	NS
PS06	2.000	1.435	0.156	0.222	0.178	0.187	0.289	*
PS24	2.364	2.016	0.437	0.313	0.421	0.427	-0.467	NS
PS25	3.273	2.358	0.469	0.507	0.195	0.162	0.180	NS
PS29	1.636	1.521	0.451	0.278	0.149	0.144	-0.501	*
PS31	2.545	2.059	0.701	0.448	0.360	0.360	-0.603	*
PS32	2.182	1.695	0.519	0.334	0.284	0.276	-0.494	*
PS36	2.545	2.079	0.617	0.437	0.277	0.275	-0.423	*
PS37	1.545	1.407	0.339	0.220	0.380	0.372	-0.391	*
PS40	1.909	1.280	0.238	0.163	0.375	0.350	-0.160	NS
PS43	2.455	1.782	0.572	0.378	0.434	0.434	-0.432	*
PS44	2.273	2.138	1.000	0.527	0.124	0.124	-0.909	*
PS47	1.545	1.300	0.242	0.176	0.555	0.527	-0.206	NS
PS51	2.000	1.445	0.365	0.253	0.412	0.411	-0.381	*
PS52	2.273	1.995	0.946	0.498	0.116	0.116	-0.897	*
CW3164	2.818	2.059	0.710	0.477	0.345	0.333	-0.505	NS
CW3189	3.273	2.592	0.799	0.552	0.234	0.235	-0.526	*
CW3248	2.909	2.221	0.807	0.510	0.277	0.272	-0.624	*
Mean	2.273	1.810	0.529	0.356	0.302	0.297	-0.441	-

Genetic diversity parameters include *Na* number of different alleles, *Ne* number of effective alleles, *Ho* observed heterozygosity, *He* expected heterozygosity, *Fst*^*a*^ *Fst* not using ENA, *Fst*^*b*^ *Fst* using ENA, and *F*_*IS*_ inbreeding coefficient = 1- (*Ho / He*). HWE indicates global test of departure from Hardy-Weinberg equilibrium. Asterisk (*) indicates a significant departure from Hardy-Weinberg equilibrium with a global test at 5% level. NS means not significant.

**Table 4:** Genetic diversity for 11 populations of *P*. *scolopendrifolia*.

Population	Sample size	*Na*	*Ne*	*Ho*	*He*	*P (%)*	*Pa*	*Ar*	*F*_*IS*_	null allele(s)
NJ	32	3.167	2.200	0.544	0.468	94	2	2.556	-0.123	PS40, PS43
MP-1	5	1.500	1.433	0.367	0.228	50	1	1.500	-0.481	-
MP-2	13	2.833	1.991	0.568	0.434	94	2	2.466	-0.279	-
HN-1	14	2.111	1.714	0.599	0.339	78	2	1.857	-0.620	-
HN-2	7	1.833	1.816	0.778	0.412	83	-	1.833	-0.867	PS25
JD-1	58	4.056	2.347	0.515	0.479	100	7	2.808	-0.057	PS6, CW3164
JD-2	11	2.722	2.104	0.540	0.448	83	2	2.502	-0.220	PS6, CW3189
GM	6	2.278	1.919	0.574	0.413	89	1	2.221	-0.347	-
WD	20	1.722	1.610	0.558	0.312	67	1	1.667	-0.678	PS25, PS29
JPN-1	8	1.500	1.500	0.500	0.250	50	4	1.500	-1.000	-
JPN-2	8	1.278	1.278	0.278	0.139	28	1	1.278	-1.000	-
Mean		2.273	1.810	0.529	0.356	74	2	2.017		

Genetic diversity parameters include *Na* number of different alleles, *Ne* number of effective alleles, *Ho* observed heterozygosity, *He* expected heterozygosity, *P* percentage of polymorphism, *Pa* private allele, *Ar* allele richness, and *F*_*IS*_ inbreeding coefficient = 1- (*Ho / He*).

Genetic diversity was evaluated for 11 populations of *P*. *scolopendrifolia* (Table 4). At the population level, the number of alleles (*Na*) across loci varied from 1.278 (JPN-2) to 4.056 (JD-1). The mean of expected alleles (*Ne*) was 1.810. While the values of observed heterozygosity (*Ho*) varied from 0.278 (JPN-2) to 0.778 (HN-2), the values of expected heterozygosity (*He*) ranged from 0.139 (JPN-2) to 0.479 (JD-1). *Ho* was greater than *He* in all populations. Regarding the percentage of polymorphism (*P*), the smallest value was found in JPN-2 (28%) and the largest value (100%) was confirmed in the population JD-1. All populations other than HN-2 had one or more private alleles. Allele richness across loci showed that JD-1 had the highest mean value of 2.808. Negative *F*_*IS*_ values were identified in all populations.

### Population genetic structure based on microsatellite markers

**Fig 1:**
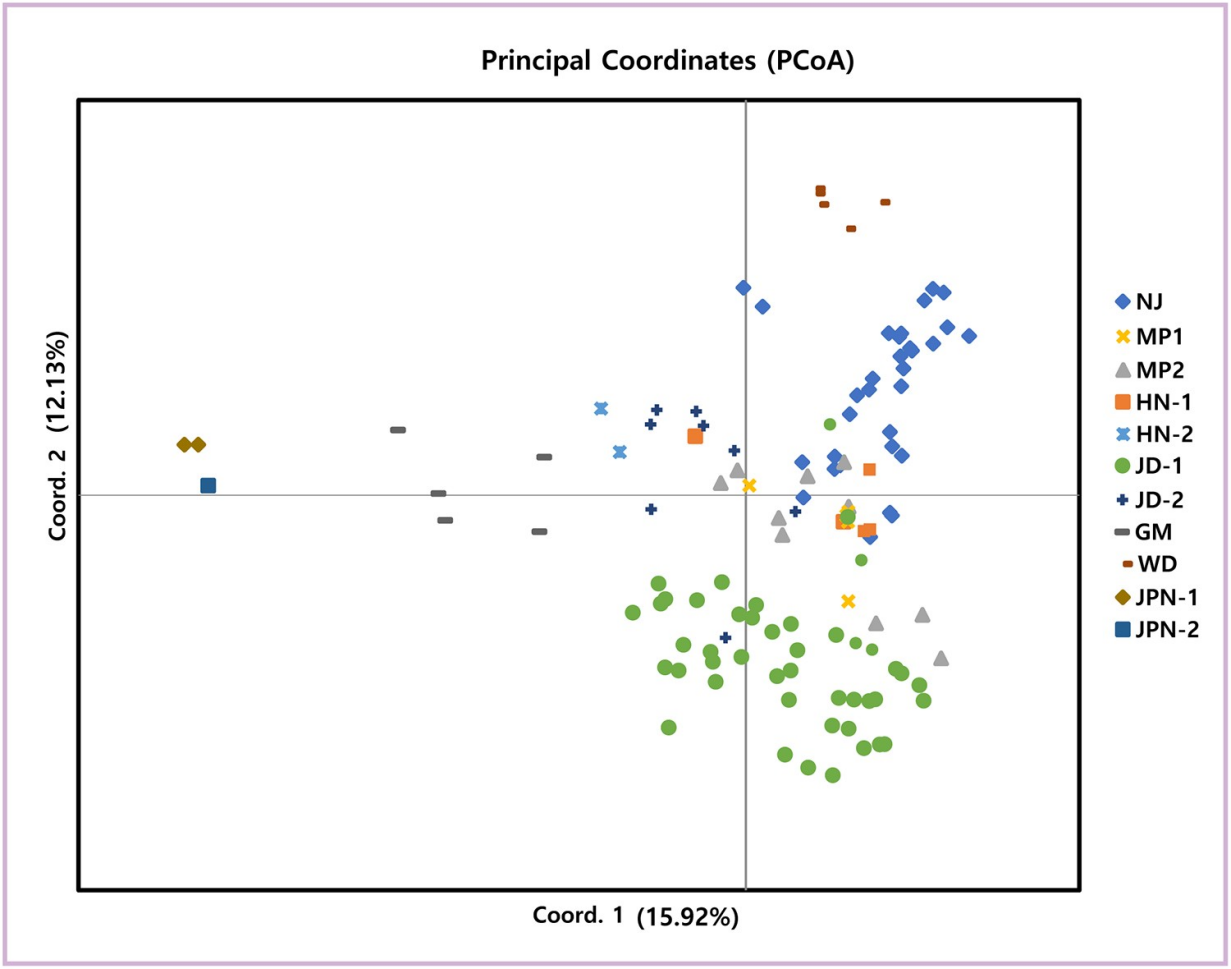
Principle coordinates analysis of variance. Principal coordinate 1 and 2 account for 15.92% and 12.13% of the variation, respectively.

**Fig 2:**
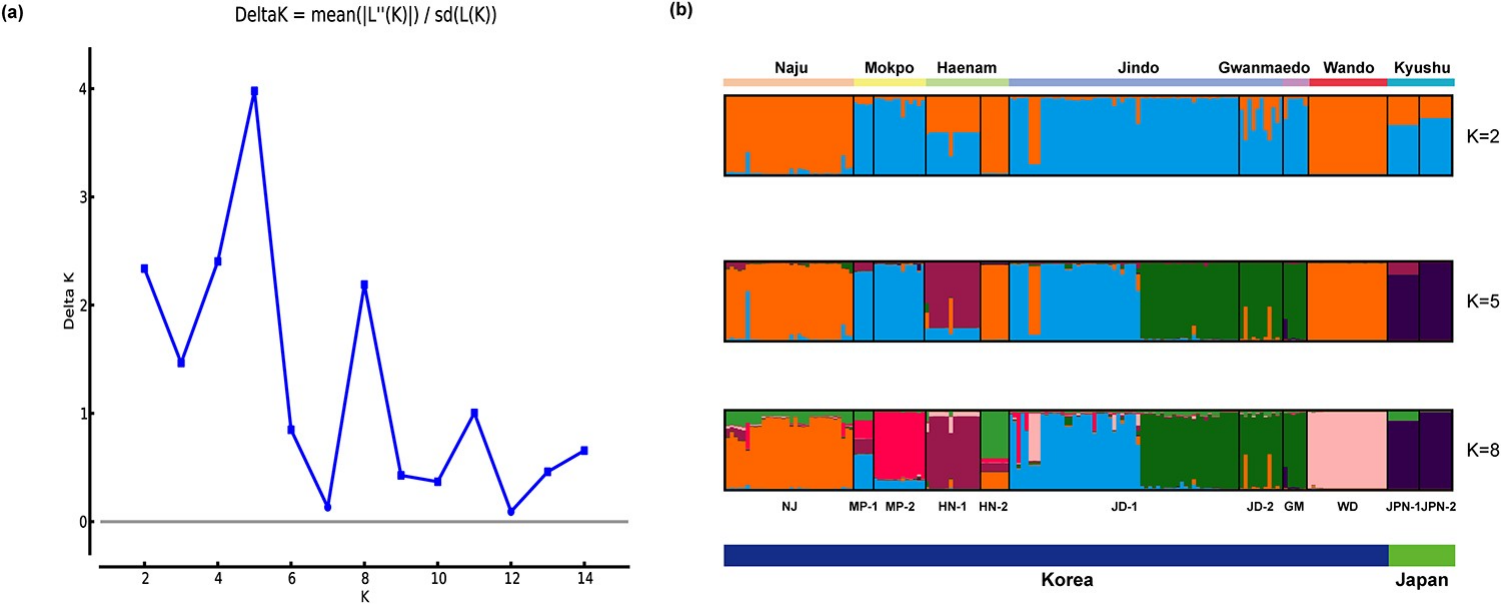
Results of STRUCTURE analyses based on microsatellite data. (a) Best K as determined by calculating ln(K) and ΔK [48] using STRUCTURE HARVESTER [49]. Best K = 5. (b) Bayesian clustering results obtained using STRUCTURE [47]. CLUMPAK [50] was used for visualization. Vertical black bar separate individuals of different populations and colors correspond to specific clusters.

PCoA results for *P*. *scolopendrifolia* populations based on 18 microsatellite markers can be seen in Fig 1. The first two principal coordinates of individual genotypes explained 28.05% of the total genetic variance (15.92% and 12.13%, respectively). Although some individuals belonging to different populations had overlapped positions (e.g., HN-1 and MP-2, and MP-2 and NJ), most of the population showed generally distinct distributions from each other. The distribution of Korean and Japanese populations were particularly different. In AMOVA analysis, 38% of the total molecular variation was attributed to among regions while 30% and 32% of the molecular variation resided among and within populations, respectively (Table 5). We conducted an additional AMOVA analysis by setting three population types (costal, inland, and island) as regions. The result detected no variance among regions while 55% and 45% of molecular variation was respectively partitioned among and within population (S4 Table). The Mantel test showed a positive correlation between genetic and geographic distances in both datasets (R^2^ = 0.5286, *P* = 0.001 using all populations; R^2^ = 0.1867, *P* = 0.001 using nine Korean populations) (S1 Fig). According to the STRUCTURE analysis, an optimal number of genetic clusters determined as ΔK reached a maximum value at K = 5. However, ΔK was also high for K = 2 and K = 8 (Fig 2). At K = 5, HN-1 consists of a population-specific genetic cluster (magenta). Although HN-1 and HN-2 are in the same region (Haenam), they showed different genetic structures. HN-2 was included in the same genetic cluster as the Wando and Naju populations. JD-1 had three genetic clusters shared with JD-2 and GM (green), and HN-2, NJ and WD (orange) as well as MP-1 and MP-2 (sky-blue). The two Japanese populations shared the same genetic cluster with each other, which was not found in any Korean populations. According to the Wilcoxon test, eight populations had a significant heterozygote excess under the TPM and SMM (*p* < 0.05), whereas nine populations showed shift mode, suggesting the occurrence of a recent bottleneck (Table 6).

**Table 5:** Result of analysis of molecular variance based on microsatellite data, showing degree of freedom (*d*. *f*.), sum of squares (SS), and percentage of variation (PV).

Source	*d*.*f*.	SS	PV (%)
Among Regions (Korea and Japan)	1	248.282	38%
Among Populations	9	781.292	30%
Within Populations	171	923.322	32%
Total	181	1952.896	100%

**Table 6:** Results of BOTTLENECK analysis conducted by the Wilcoxon test method and mode-shift test based on microsatellite data.

Population	Wilcoxon test		Mode shift
	TPM	SMM	
NJ	0.00052*	0.11234	normal L-shaped
MP-1	0.00488*	0.00488*	shifted mode
MP-2	0.12175	0.53674	shifted mode
HN-1	0.01227*	0.03381*	shifted mode
HN-2	0.00002*	0.00002*	shifted mode
JD-1	0.36686	0.87690	normal L-shaped
JD-2	0.00168*	0.03186*	shifted mode
GM	0.0145*	0.04163*	shifted mode
WD	0.00024*	0.00024*	shifted mode
JPN-1	0.00098*	0.00098*	shifted mode
JPN-2	0.01563*	0.01563*	shifted mode

For Wilcoxon test, the values represent probability of one-tailed probabilities for heterozygote excess and * indicates *p* < 0.05.

### Chloroplast haplotype frequency and distribution

**Fig 3:**
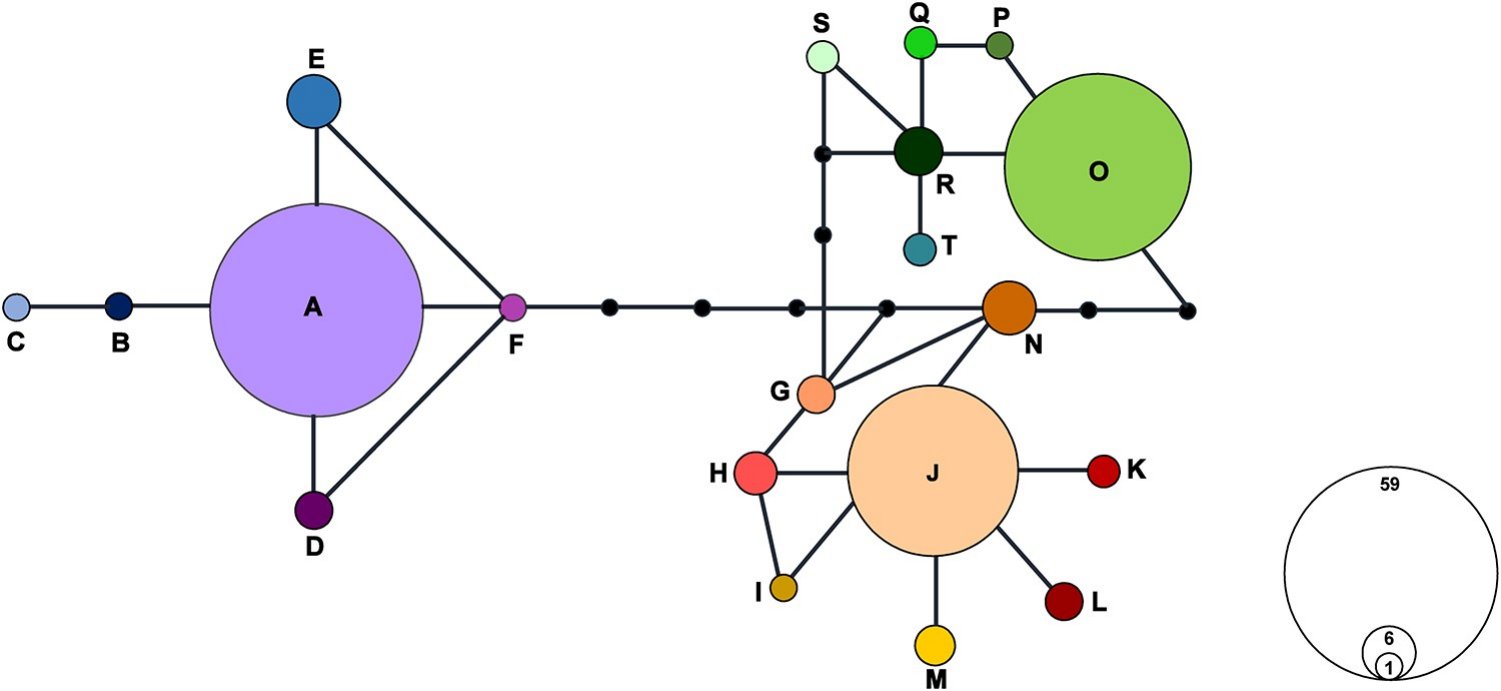
Haplotype network constructed using TCS software based on chloroplast DNA data. Twenty haplotypes were found in *P*. *scolopendrifolia*. Each haplotype is represented by a circle, and the size of each circle is proportional to the number of individuals with that haplotype. Each line connecting two haplotypes represents a single mutation, and dots indicate hypothetical missing haplotypes.

**Fig 4:**
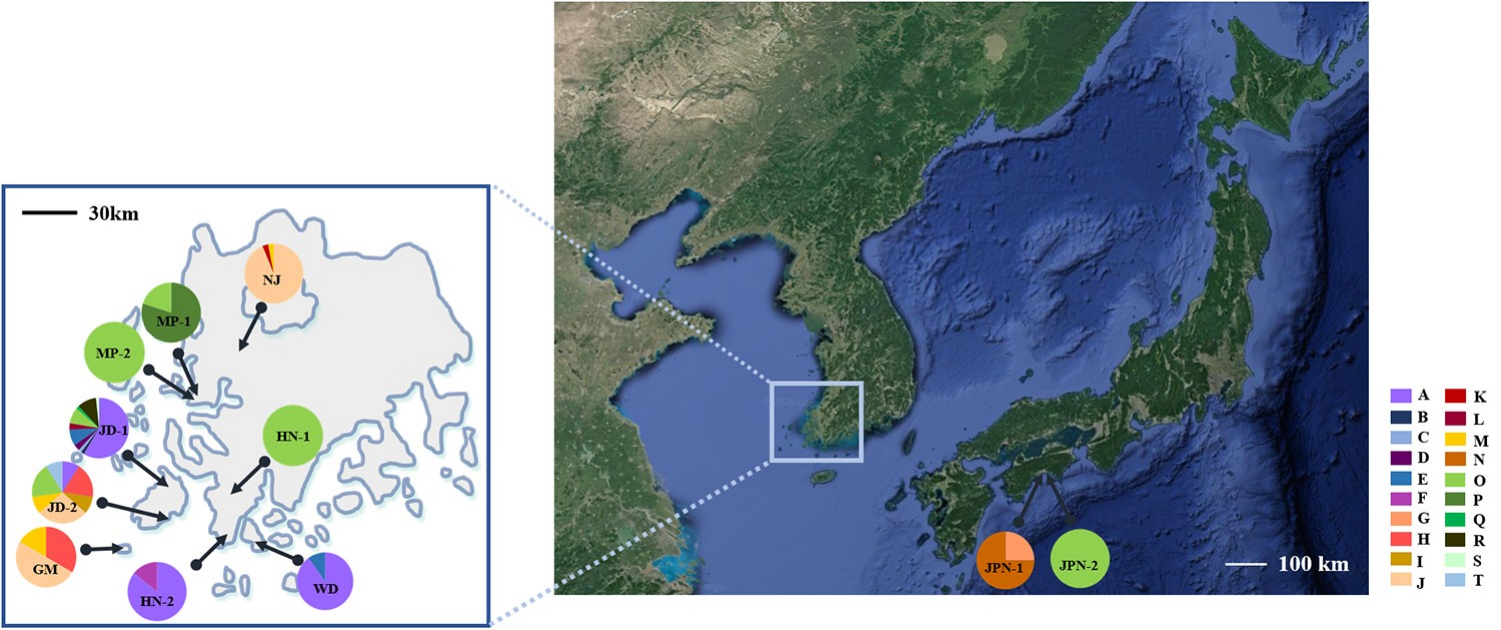
Distribution of *P*. *scolopendrifolia* haplotype based on chloroplast DNA data.

The sequences of *acc*D-*psb*I, *pet*A-*psb*J, *pet*L-*psb*E and *rps*12-*rpl*20 with polymorphic sites were successfully amplified and sequenced in 182 individuals from the 11 populations. The total length of the combined alignment was 2,370 bp. A total of 20 chloroplast haplotypes (A-T) were identified from this study (Table 1 and Fig 3). The most abundant haplotype was A (32%, 59 accessions), followed by O (25%, 46 accessions) and J (20%, 36 accessions). Of the 20 haplotypes, six (A, E, H, J, M, and O) were shared in two or more populations. However, nine haplotypes (B, C, F, I, K, P, Q, S, and T) were shown in only one individual and five haplotypes (D, G, L, N, and R) were population specific. The population with the most haplotype was JD-1 while MP-2 and HN-1 had only one haplotype each. Star-like network patterns were formed around the major haplotypes A and J (Fig 3). The genealogical relationships among haplotypes based on the TCS analysis revealed two major groups. The first one (group I) included six haplotypes (A-F), which could be found in four populations (HN-2, JD-1, JD-2, and WD), while the other (group II) was comprised of the remaining haplotypes. All populations other than HN-2 and WD include haplotypes from group II. The haplotypes were shared between the populations distributed in the same region (MP-1 and MP-2, and JD-1 and JD-2) or geographically close populations (HN-2 and WD). Although HN-1 and HN-2 were in the same region on the administrative district, they had different haplotypes. Notably, haplotypes J and M of population NJ were shared with JD-2 and GM, yet these haplotypes were not identified in other populations between NJ and Jindo region (Fig 4).

### Genetic diversity and structure based on chloroplast DNA data

Gene diversity of populations ranged from 0.0000 (MP-2, HN-1, and JPN-2) to 0.9091 (JD-2). Nucleotide diversity was highest in JD-1 while MP-2, HN-1, and JPN-2 were the lowest as having the value of 0.0000. Overall, genetic diversities were thoroughly evaluated in the Jindo (JD-1 and JD-2) region given its relatively large population size. Values of genetic diversity were high in Korea compared to those in Japan. Tajima’s *D* and Fu’s *Fs* for neutrality of selection pressure revealed that NJ had the only negative and significant values (Tajima’s *D* = -1.5037, *Fs* = -2.4372, *p* < 0.05) (Table 7). AMOVA analysis showed significant genetic variation in among population within regions (Korea and Japan) (Table 8). According to the AMOVA using three population types as regions, most genetic variation occurred among populations within regions (63.06%) (S4 Table).

**Table 7:** Estimation of gene diversity, nucleotide diversity, and neutrality of 11 populations and two countries based on chloroplast DNA data.

Population	Gene diversity	Nucleotide diversity	Neutrality			
			Tajima's *D*	Tajima's *D p*-value	Fu's *Fs*	*Fs p*-value
NJ	0.123±0.0777	0.000053±0.000093	-1.5037	0.0320	-2.4372	0.0030
MP-1	0.4000±0.2373	0.000169±0.000215	-0.8165	0.3050	0.0902	0.3480
MP-2	0.0000±0.0000	0.0000±0.0000	0.0000	1.0000	0.0000	NA
HN-1	0.0000±0.0000	0.0000±0.0000	0.0000	1.0000	0.0000	NA
HN-2	0.2857±0.1964	0.000128±0.000175	-1.0062	0.2320	-0.0947	0.2270
JD-1	0.6382±0.0674	0.001886±0.001045	1.1351	0.9120	1.6853	0.7690
JD-2	0.9091±0.0656	0.001695±0.001034	-0.7022	0.2620	-0.8943	0.2730
GM	0.7333±0.1552	0.000366±0.000341	-0.0500	0.4380	-0.4268	0.1900
WD	0.1895±0.1081	0.000080±0.000118	-0.5916	0.2340	-0.0966	0.2420
JPN-1	0.4286±0.1687	0.000181±0.000206	0.3335	0.8200	0.5363	0.4430
JPN-2	0.0000±0.0000	0.0000±0.0000	0.0000	1.0000	0.0000	NA
Country						
Korea	0.7743±0.0188	0.002299±0.001231	1.9362	0.9760	0.7517	0.6560
Japan	0.6333±0.0737	0.000774±0.000525	1.6295	0.9540	2.5518	0.8910

**Table 8:** Results of analysis of molecular variance (AMOVA) in *P*. *scolopendrifolia* based on chloroplast DNA data, showing degree of freedom (*d*. *f*.), sum of squares (SS), variance components (VC), and percentage of variation (PV).

Source of variation	*d*. *f*.	SS	VC	PV (%)	Fixation indices	*p* value
2 regions (Korea and Japan)						
Among regions	1	21.741	-0.0197	-0.66	F_CT_ = -0.00656	0.44575±0.01448
Among populations within regions	9	306.778	2.10884	70.19	F_SC_ = 0.69734	0.00000±0.00000
Within populations	171	156.514	0.91529	30.46	F_ST_ = 0.69535	0.00000±0.00000
Total	181	485.033	3.00442			

## Discussion

### Development of microsatellite markers for population genetic study

This study reported microsatellite markers newly developed in *Pelatantheria scolopendrifolia*, one of highly threatened and endangered orchid species in Korea. A total of 15 polymorphic microsatellite markers were successfully amplified in all individuals from 11 populations, showing observed heterozygosity values from 0.156 to 1.000 (mean *Ho* = 0.481) and expected heterozygosity from 0.119 to 0.527 (mean *He* = 0.325). Although the values of heterozygosity were similar to or lower than those of other orchid species, for example, *Caladenia huegelii* (mean *Ho* = 0.551 and *He* = 0.690) [58] and *Cypripedium kentuckiense* (mean *Ho* = 0.436 and *He* = 0.448) [20], the presence of private alleles and the average number of alleles per locus of 5.13 suggest that the markers were appropriate for evaluating genetic diversity in this study. We could not verify the transferability of microsatellite markers into closely related species due to there being only one genus one species in Korea. Even though microsatellite markers were known to have species-specific characterization, there are many studies that successfully apply microsatellite markers into a closely related species. For example, this has been shown in *Pinus resinosa* (Pinaceae) [59], *Allium sativum* (Lilliaceae) [60], *Artocarpus altilis* (Moraceae) [61], and *Prunus mongolica* (Rosaceae) [62]. Therefore, these sets of microsatellite markers will likely prove useful in future population genetic studies related to the genus *Pelatantheria* as well as *P*. *scolopendrifolia* specially.

### Patterns of genetic diversity

Since species with small population size or limited geographic range are vulnerable to reduced genetic diversity, studies on the genetic diversity of these species are of particular importance for conservation [63–65]. To assess genetic diversity of the endangered orchid *Pelatantheria scolopendrifolia*, we used two types of molecular markers, 18 microsatellite markers and four non-coding cpDNA markers. In this study, we found that the values of observed heterozygosity based on microsatellite genotyping dataset ranged from 0.278 to 0.778 with an average value of 0.529. The values of expected heterozygosity (mean *Ho* = 0.356) were all lower than those of observed heterozygosity, suggesting heterozygosity excess. The mean *He* and *Ho* values observed in *P*. *scolopendrifolia* are lower when compared to other endangered or endemic orchids. For example, *Caladenia huegelii* had a mean *He* = 0.69 and mean *Ho* = 0.551 [58], and *Cymbidium tortisepalum* had a mean *He* = 0.653 and mean *Ho* = 0.619 [21]. On the other hand, values of expected heterozygosity of *P*. *scolopendrifolia* were similar to *Gastrodia elata* which was evaluated as having low genetic variation [66]. Rare and endemic species as well as geographically restricted species are expected to have lower levels of genetic diversity than widespread species [67–70]. Compared to these results, genetic diversity of *P*. *scolopendrifolia* is lower to the mean *He* and *Ho* values reported for narrow and regional ranged species [69]. Given that genetic diversity is deeply influenced by the life cycle, distribution range, the pattern of reproduction, and gene flow [71–73], life cycle can be ruled out as a cause of low genetic diversity due to the longevity of this species. In accordance with the vulnerability of small populations to genetic drift [1] and subsequent increases of homozygosity [74], these factors likely play a large role in the low genetic diversity of both Korean and Japanese *P*. *scolopendrifolia* populations. Historical demographic events, such as founder effects, bottlenecking, population expansion and contraction, and habitat fragmentation, can all impact genetic diversity and structure [63, 75, 76]. The previous genetic study of *P*. *scolopendrifolia* using allozymes suggested that bottleneck and founder effects are possible causes for extremely low levels of allozyme variation [2]. In this study, evidence of bottleneck effects were found via BOTTLENECK analysis (Table 6). Nine out of 11 populations showed a distribution of alleles with a shifted mode, suggesting the presence of a recent bottleneck. In contrast, the large populations NJ and JD-1 indicated a normal L-shaped mode, as expected in the absence of a recent bottleneck. Also, in spite of high haplotype diversity, gene and nucleotide diversity values are low (Fig 3, Table 7), indicating rapid population expansion [76]. In Tajima’s *D* and Fu’s *Fs* test to determine the presence of the population expansion, six populations showed negative values on Tajima’s *D* and Fu’s *Fs*, but only NJ was significant (*p* < 0.05), corroborating the aforementioned result. The star-like shape of the haplotype network also supported this assumption. Therefore, this study suggests that demographic events including bottlenecks and population expansion have played important roles in reducing genetic diversity of *P*. *scolopendrifolia*. Reproductive pattern may also contribute to this low genetic diversity. Mating system can be very influential, as reproduction through outcrossing is known to lead to a high level of genetic diversity [77]. Chung et al. [2] reported that *P*. *scolopendrifolia* is self-compatible, while Son et al. [35] came to the opposite conclusion based on the correlation between the pollinator visits (male Megachile bee, *Megachile yasumatsui*) and fruit set. The diversity of the populations MP-2, HN-2, and WD confirmed pollinator visits were not significantly higher than the populations without a pollinator. Furthermore, gene diversity, nucleotide diversity, and haplotype diversity based on cpDNA sequences were unable to reveal any relationship with the presence or absence of a pollinator. These results are contrary to the population genetic theory which generally holds that the genetic diversity of outcrossing species tends to be higher [69]. This may be because *P*. *scolopendrifolia* can be reproduced through clonal ramets in addition to outcrossing. Vegetative reproduction is one of the causes low genetically variation [78, 79]. Conversely, the patterns of allele richness and private allele abundance in *P*. *scolopendrifolia* demonstrate that genetic diversity tends to be higher in larger population sizes due to the higher likelihood of maintaining a robust genetic pool. The gene flow is another factor to explain low genetic diversity. In general, pollen and seed dispersal are important for maintaining gene flow. Gene flow between populations prevent from differentiation and increase genetic diversity between populations. Despite the presence of pollinators, the reason for low genetic diversity may be related to the behavior of the pollinator itself. It is not known how far Megachile bees fly, but male bees typically carry pollen farther distances than female bees [80], and Euglossine bees can return to their nest from as far 23 km in tropical rain forest [81]. The distance between the populations of *P*. *scolopendrifolia* with and without pollinators is approximately 30 km. Therefore, it is probably that little to no pollinator activity occurs between populations of *P*. *scolopendrifolia*. Based on these facts, it is inferred that gene flow by pollen dispersal is weak. This is consistent with PCoA and STRUCTURE results indicating the population differentiation (Figs 1 and 2). However, the haplotypes shared between or among populations may provide evidence of gene flow through seed dispersal by wind (Fig 4). The fruits of *P*. *scolopendrifolia* are matured during late summer and autumn. Because Korea has a monsoon climate, winds from the southeast or south blow in summer and winds from the northwest blow in winter. In the fall, there is no distinct wind direction as it corresponds to the transitional period of the two monsoons. Therefore, the direction of seed dispersal can be random and haplotype diversity might not distribute in one direction. Various factors, including small population size, demographic history, asexual reproduction, and low gene flow, likely contribute to the low levels genetic diversity seen in *P*. *scolopendrifolia*, as well as causing significant LD values (S1 Table).

### Population differentiation and genetic structure

The mean *Fst* value (0.299, S2 Table) among populations measured by microsatellite markers was lower than the *Fst* value (0.899) based on allozymes [2]. Comparison of population differentiation generated via different types of markers might be not appropriate; however, the mean *Fst* of *P*. *scolopendrifolia* is higher than that of long-lived perennial, narrow-ranged, and outcrossing species (0.19, 0.23, and 0.22, respectively) [69]. This result was supported by a PCoA based on microsatellite data (Fig 1) and AMOVA analysis based on cpDNA data, with both showing that the majority of genetic variation existed among populations within regions (Table 8, S4 Table). This paints a picture of differentiation among populations, that is consistent with the previous study [2]. These results might be explained by limited gene flow among populations and their isolation. The results of the Mantel test also indicate positive correlations between geographic and genetic distances, supporting population differentiation by isolation (S1 Fig). Significant pairwise genetic differentiation was detected between NJ and JD-1/JD-2 (S2 Table). The two populations are approximately 70 km apart; however, they had the smallest different in *Fst* values, further supported by genetically similarity based on their haplotype distributions (Fig 4). Although it is not supported by STRCUTURE analysis, haplotypes J and M were shared with each other. This may additionally raise the possibility of seed dispersal from JD-1 or JD-2 to NJ that has recently undergone population expansion. Nonetheless, a more thorough and clear understanding of these genetic pattern will require future study involving more rigorous comparison of NJ and the Jindo populations. The Bayesian analysis grouped the populations into 5 clusters (K = 5; Fig 2). The admixture structure existed only weakly, and most populations showed a simple structure with geographically close populations (e.g., GM and JD-1/JD-2) having the same structure. This is also somewhat consistent with the patterns of shared haplotypes between geographically close populations other than JPN-1 and JPN-2 (Fig 4). Although identical genetic structure and shared haplotypes indicate pollen or seed dispersal between or among populations, bottleneck or founder event and geological barriers to prevent gene flow could be factors to affect simple genetic structures (Fig 2) and population-specific haplotypes (Fig 4). Notably, JD-1 is composed of three genetic clusters, indicating the presence of three genetically distinct subpopulations. Therefore, the founders of this population either had substantially different genetic compositions, or each subpopulation had a different origin.

### Haplotype distribution and dispesal of *P*. *scolopendrifolia*

According to Qian and Ricklefs [82], during periods of glacial maxima (about 18,000 years ago) in the Pleistocene China, Korea, and Japan were connected by continental shelf. Simulated vegetation maps of East Asia during the last glacial maximum [82, 83] showed vegetation distributions among East China, the Korean Peninsula and Japan that coincide with the current distribution pattern of *P*. *scolopendrifolia*. This raises the possibility that the influx of *P*. *scolopendrifolia* to Korea was not only from China, but also from Japan. Three major haplotypes (A, J, and O) in the haplotype network support this notion. In addition, founder events may also have influenced the haplotype distribution. After the influx of *P*. *scolopendrifolia* from China or Japan, genetic drift related to founder effects or bottlenecks in the process of dispersal from southern to northern populations would have contributed to the low level of genetic diversity and simple population structure. In this process, the Jindo populations (JD-1 and JD-2) with relatively high genetic variation might be situated as a geographic center of seed dispersal. However, after colonization, factors such as excessive collection, asexual reproduction, geographic isolation, and the loss of genetic diversity due to low gene flow provide strong reasons for the absence of a specific pattern in the haplotype distribution map (for example, the relevance of distance and genetic structure).

### Implications for conservation

*P*. *scolopendrifolia* is categorized as an endangered species according to IUCN Red List criteria: B2b(iii,iv)c(iii,iv,v). The species is also listed as vulnerable in Japan following ‘Japanese Red Lists’ (https://www.nationalredlist.org). Therefore, the development of policies for the conservation of *P*. *scolopendrifolia* is essential. Fortunately, since 1998, it has been legally protecting *P*. *scolopendrifolia* in Korea. Information on genetic diversity and population genetic structure, as well as ecological knowledge, should be considered when developing and designing conservation strategies for species or populations. Subsequently, this study provides several suggestions. First, population size must be stabilized. Most of the populations were not large, and MP-2, HN-1, JD-1, and JD-2 are easily accessible along the trail, leaving them vulnerable to illegal collection. Therefore, periodic monitoring and strict prohibition of illegal collection seem necessary. Second, the establishment of more efficient conservation strategies will require more extensive research on *P*. *scolopendrifolia* ecology. Han [33] reported that the lichen *Ramalina intestiniformis* was commonly found in association with all populations of *P*. *scolopendrifolia*. Though this relationship is not well understood, it appears related to strategies for preserving or obtaining moisture. Factors such as these can be used to create more preferable environments for *P*. *scolopendrifolia* reintroduction, thus increasing the success rate of settlement. Lastly, genetic diversity and structure should be carefully considered. Genetic diversity was evaluated as relatively low in this study as a result of low diversity in allozyme data [2]. However, the existence of population-specific private alleles and haplotypes, and the unique genetic structures of population can guide the selection of the most ideal population from which to source individuals for reintroduction to a specific location. Realizing this will require the combination of seed collection from each population and ex-situ conservation. In addition, Son et al. [34, 35] confirmed the existence of pollinators and revealed the relationship between pollinator visits and fruit set. Given that self- incompatibility and need of biotic pollinator for pollination, human-mediated pollination can subsequently be used as a strategy to increase the fruit set rate and genetic diversity. Pollinator protection strategies such as pollinator habitat enhancement and pest management are also necessary due to the local distribution of the pollinator.

## Conclusions

In this study, we newly developed 15 polymorphic microsatellite markers of *P*. *scolopendrifolia* and provided information related to genetic diversity and population structure based on microsatellite and cpDNA data. Low levels of genetic diversity and population differentiation were observed. In addition, the identification of the private alleles and population-specific haplotypes, further enhances the need for conservation. However, it was not possible to find a connection between genetic structure or haplotype distribution and specific factors such as the presence of pollinator. Various causes such as small population size, demographic events, clonal reproduction, and low gene flow all likely have role in shaping the observed genetic patterns of *P*. *scolopendrifolia*. Lastly, taking these patterns into account informed several suggestions for the development of future conservation strategies.

## Supporting information

S1 Table Linkage disequilibrium over all loci and populations based on microsatellite data. + and − indicate significant and non-significant level at *p* < 0.05.
(DOCX)

S2 Table *F*_*ST*_ values among pairs of populations. Probability values are all under the 0.005.
(DOCX)

S3 Table Variable sites found *in Pelatantheria scolopendrifolia* identifying 20 haplotypes. Question mark means missing data. * indicates the sequencing modification. T and A on 633 site were originally TCTTAATAT and 9 bp deletion, respectively; On 665 site, 1 bp deletion character was coded as A; T and C on 775 site stand for TAAGG and CCTTA, respectively.
(DOCX)

S4 Table Analyses of molecular variance using microsatellite and chloroplast DNA data. (a) Result based on microsatellite data. (b) Result based on chloroplast DNA data. For this analysis, three population types: coastal, inland, and island, were set as region. Regions: inland (NJ, HN-1), coastal (MP-1, MP-2, and HN-2), and island (JD-1, JD-2, WD, GM, JPN-1, and JPN-2).
(DOCX)

S1 Fig Relationship between pairwise genetic distance and geographic distance of *Pelatantheria scolopendrifolia* populations. (a) all populations. (b) Korean populations. The results of the Mantel test indicate the positive correlation between genetic and geographic distances was significant. Note: *P* = 0.001 for all correlations.
(TIF)

## References

[1] Ellstrand NC, Elam DR. Population genetic consequences of small population size: implications for plant conservation. Annu Rev Ecol Syst. 1993;24: 217–242.

[2] Chung MY, Park CW, Chung MG. Extremely low levels of allozyme variation in southern Korean populations of the two rare and endangered lithophytic or epiphytic *Bulbophyllum drymoglossum* and *Sarcanthus scolopendrifolius* (Orchidaceae): implications for conservation. Biodivers Conserv. 2007;16: 775–786.

[3] Liu F, Hong Z, Xu D, Jia H, Zhang N, Liu X, et al Genetic diversity of the endangered *Dalbergia odorifera* revealed by SSR markers. Forests. 2019;10: 225.

[4] Glover BJ, Abbott RJ. Low genetic diversity in the Scottish endemic *Primula scotica* Hook. New Phytol. 1995;129: 147–153. 10.1111/j.1469-8137.1995.tb03018.x 33874421

[5] Young AG, Brown AHD, Zich FA. Genetic structure of fragmented populations of the endangered daisy *Rutidosis leptorrhynchoides*. Conserv Biol. 1999;13: 256–265.

[6] Zhai SH, Yin GS, Yang XH. Population genetics of the endangered and wild edible plant *Ottelia acuminata* in southwestern China using novel SSR markers. Biochem Genet. 2018;56: 235–254. 10.1007/s10528-018-9840-2 29350309

[7] Johnson SD. Insect pollination and floral mechanisms in South African species of *Satyrium* (Orchidaceae). Plant Syst Evol. 1997;204: 195–206.

[8] Johnson SD, Linder HP, Steiner KE. Phylogeny and radiation of pollination systems in *Disa* (Orchidaceae). Am J Bot. 1998;85: 402–411. 21684924

[9] Davies KL, Turner MP. Morphology of floral papillae in *Maxillaria* Ruiz & Pav. (Orchidaceae). Ann Bot. 2004;93: 75–86. 10.1093/aob/mch007 14630691 PMC4242261

[10] Szendrák E. Asymbiotic in vitro seed germination, micropropagation and scanning electron microscopy of several temperate terrestrial orchids (Orchidaceae). Ph.D. Thesis, University of Nebraska. 1997. https://digitalcommons.unl.edu/agronhortdiss/1/

[11] Dijk E, Willems JH, Van Andel J. Nutrient responses as a key factor to the ecology of orchid species. Acta Bot Neerl. 1997;46: 339–363.

[12] Rasmussen HN, Dixon KW, Jersáková J, Těšitelová T. Germination and seedling establishment in orchids: a complex of requirements. Ann Bot. 2015;116: 391–402. 10.1093/aob/mcv087 26271118 PMC4549959

[13] Borba EL, Felix JM, Solferini VN, Semir J. Fly-pollinated *Pleurothallis* (Orchidaceae) species have high genetic variability: evidence from isozyme markers. Am J Bot. 2001;88: 419–428. 11250819

[14] Chung MY, López-Pujol J, Chung MG. Low genetic diversity in marginal populations of *Bletilla striata* (Orchidaceae) in southern Korea: Insights into population history and implications for conservation. Biochem Syst Ecol. 2013;46: 88–96.

[15] Chung MY, López-Pujol J, Maki M, Moon MO, Hyun JO, Chung MG. Genetic variation and structure within 3 endangered *Calanthe* species (Orchidaceae) from Korea: inference of population-establishment history and implications for conservation. J Hered. 2013;104: 248–262. 10.1093/jhered/ess088 23125404

[16] Duffy KJ, Scopece G, Cozzolino S, Fay MF, Smith RJ, Stout JC. Ecology and genetic diversity of the dense-flowered orchid, *Neotinea maculata*, at the centre and edge of its range. Ann Bot. 2009;104: 507–516. 10.1093/aob/mcn200 18940852 PMC2720646

[17] Pillon Y, Qamaruz-Zaman F, Fay MF, Hendoux F, Piquot Y. Genetic diversity and ecological differentiation in the endangered fen orchid (*Liparis loeselii*). Conserv Genet. 2007;8: 177–184.

[18] Jacquemyn H, Brys R, Adriaens D, Honnay O, Roldán-Ruiz I. Effects of population size and forest management on genetic diversity and structure of the tuberous orchid *Orchis mascula*. Conserv Genet. 2009;10: 161–168.

[19] Qian X, Wang CX, Tian M. Genetic diversity and population differentiation of *Calanthe tsoongiana*, a rare and endemic orchid in China. Int J Mol Sci. 2013;14: 20399–20413. 10.3390/ijms141020399 24129175 PMC3821621

[20] Pandey M, Richards M, Sharma J. Microsatellite-based genetic diversity patterns in disjunct populations of a rare orchid. Genetica. 2015;143: 693–704. 10.1007/s10709-015-9867-9 26481007

[21] Zhao Y, Tang M, Bi Y. Nuclear genetic diversity and population structure of a vulnerable and endemic orchid (*Cymbidium tortisepalum*) in Northwestern Yunnan, China. Sci Hortic (Amsterdam). 2017;219: 22–30.

[22] Brown SM, Hopkins MS, Mitchell SE, Senior ML, Wang TY, Duncan RR, et al Multiple methods for the identification of polymorphic simple sequence repeats (SSRs) in *sorghum* [*Sorghum bicolor* (L.) Moench]. Theor Appl Genet. 1996;93: 190–198. 10.1007/BF00225745 24162217

[23] Arnold C, Rossetto M, McNally J, Henry RJ. The application of SSRs characterized for grape (*Vitis vinifera*) to conservation studies in Vitaceae. Am J Bot. 2002;89: 22–28. 10.3732/ajb.89.1.22 21669708

[24] Lorenz-Lemke AP, Togni PD, Mäder G, Kriedt RA, Stehmann JR, Salzano FM, et al Diversification of plant species in a subtropical region of eastern South American highlands: a phylogeographic perspective on native *Petunia* (Solanaceae). Mol Ecol. 2010;19: 5240–5251. 10.1111/j.1365-294X.2010.04871.x 21040052

[25] Mingeot D, Husson C, Mertens P, Watillon B, Bertin P, Druart P. Genetic diversity and genetic structure of black alder (*Alnus glutinosa* [L.] Gaertn) in the Belgium-Luxembourg-France cross-border area. Tree Genet Genomes. 2016;12: 24.

[26] Kim SH, Cho MS, Li P, Kim SC. Phylogeography and ecological niche modeling reveal reduced genetic diversity and colonization patterns of skunk cabbage (*Symplocarpus foetidus*; Araceae) from glacial refugia in eastern North America. Front Plant Sci. 2018;9: 648 10.3389/fpls.2018.00648 29872442 PMC5972301

[27] Mummenhoff K, Brüggemann H, Bowman JL. Chloroplast DNA phylogeny and biogeography of *Lepidium* (Brassicaceae). Am J Bot. 2001;88: 2051–2063. 21669637

[28] Lee C, Kim SC, Lundy K, Santos-Guerra A. Chloroplast DNA phylogeny of the woody *Sonchus* alliance (Asteraceae: Sonchinae) in the Macaronesian Islands. Am J Bot. 2005;92: 2072–2085. 10.3732/ajb.92.12.2072 21646124

[29] Bayer RJ, Mabberley DJ, Morton C, Miller CH, Sharma IK, Pfeil BE. A molecular phylogeny of the orange subfamily (Rutaceae: Aurantioideae) using nine cpDNA sequences. Am J Bot. 2009;96: 668–685. 10.3732/ajb.0800341 21628223

[30] Provan J, Powell W, Hollingsworth PM. Chloroplast microsatellites: new tools for studies in plant ecology and evolution. Trends Ecol Evol. 2001;16: 142–147. 10.1016/s0169-5347(00)02097-8 11179578

[31] National Institute of Biological Resources. Korean Red List of Threatened Species. 2nd ed Incheon: Jisungsa; 2014.

[32] Frankham R. Conservation genetics. Annu Rev Genet. 1995;29: 305–327. 10.1146/annurev.ge.29.120195.001513 8825477

[33] Han JE. A Research on the Genetic Diversity of Neofinetia falcata and Cleisostoma scolopendrifolium (Orchidaceae, Tribe Vandeae) with Biomonitoring Research using Lichens. Ph.D. Thesis, Inha University. 2017. https://academic.naver.com/openUrl.naver?doc_id=291511184&linkType=doclink

[34] Son HD, Im HT, Choi SW. Study on the pollinator and pollination mechanism of an endangered orchid species, *Sarcanthus scolopendrifolius* Makino (Orchidaceae) in Korea. Korean J Apic. 2017;32: 199–203.

[35] Son HD, Im HT, Choi SW. Pollination of *Cleisostoma scolopendrifolium* (Orchidaceae) by megachilid bees and determinants of fruit set in southern South Korea. J Ecol Environ. 2019;43: 3.

[36] Kearse M, Moir R, Wilson A, Stones-Havas S, Cheung M, Sturrock S, et al Geneious Basic: an integrated and extendable desktop software platform for the organization and analysis of sequence data. Bioinformatics. 2012;28: 1647–1649. 10.1093/bioinformatics/bts199 22543367 PMC3371832

[37] Thiel T, Michalek W, Varshney R, Graner A. Exploiting EST databases for the development and characterization of gene-derived SSR-markers in barley (*Hordeum vulgare* L.). Theor Appl Genet. 2003;106: 411–422. 10.1007/s00122-002-1031-0 12589540

[38] Untergasser A, Cutcutache I, Koressaar T, Ye J, Faircloth BC, Remm M, et al Primer3—new capabilities and interfaces. Nucleic Acids Res. 2012;40: e115 10.1093/nar/gks596 22730293 PMC3424584

[39] Shaw J, Lickey EB, Schilling EE, Small RL. Comparison of whole chloroplast genome sequences to choose noncoding regions for phylogenetic studies in angiosperms: the tortoise and the hare III. Am J Bot. 2007;94: 275–288. 10.3732/ajb.94.3.275 21636401

[40] Van Oosterhout C, Hutchinson WF, Wills DP, Shipley P. MICRO-CHECKER: software for identifying and correcting genotyping errors in microsatellite data. Mol Ecol Notes. 2004;4: 535–538.

[41] Dempster AP, Laird NM, Rubin DB. Maximum likelihood from incomplete data via EM algorithm. J R Stat Soc Ser B Stat Methodol. 1977;39: 1–22.

[42] Chapuis MP, Estoup A. Microsatellite null alleles and estimation of population differentiation. Mol Biol Evol. 2007;24: 621–631. 10.1093/molbev/msl191 17150975

[43] Peakall R, Smouse PE. GENALEX 6: genetic analysis in Excel. Population genetic software for teaching and research. Mol Ecol Notes. 2006;6: 288–295. 10.1093/bioinformatics/bts460 PMC3463245 22820204

[44] Goudet J. FSTAT (version 1.2): a computer program to calculate F-statistics. J Hered. 1995;86: 485–486.

[45] Rousset F. GENEPOP’ 007: a complete re-implementation of the GENEPOP software for Windows and Linux. Mol Ecol Res. 2008;8: 103–106. 10.1111/j.1471-8286.2007.01931.x 21585727

[46] Mantel N. The detection of disease clustering and a generalized regression approach. Cancer Research. 1967; 27: 209–220. 6018555

[47] Pritchard JK, Stephens M, Donnelly P. Inference of population structure using multilocus genotype data. Genetics. 2000;155: 945–959. 10835412 10.1093/genetics/155.2.945 PMC1461096

[48] Evanno G, Regnaut S, Goudet J. Detecting the number of clusters of individuals using the software STRUCTURE: a simulation study. Mol Ecol. 2005;14: 2611–2620. 10.1111/j.1365-294X.2005.02553.x 15969739

[49] Earl DA, vonHoldt BM. STRUCTURE HARVESTER: a website and program for visualizing STRUCTURE output and implementing the Evanno method. Conserv Genet Resour. 2012;4: 359–361.

[50] Kopelman NM, Mayzel J, Jakobsson M, Rosenberg NA, Mayrose I. CLUMPAK: a program for identifying clustering modes and packaging population structure inferences across K. Mol Ecol Resour. 2015;15: 1179–1191. 10.1111/1755-0998.12387 25684545 PMC4534335

[51] Piry S, Luikart G, Cornuet JM. BOTTLENECK: a program for detecting recent effective population size reductions from allele data frequencies. J Hered. 1999;90: 502–503.

[52] Templeton AR, Crandall KA, Sing CF. A cladistic analysis of phenotypic associations with haplotypes inferred from restriction endonuclease mapping and DNA sequence data. III. Cladogram estimation. Genetics. 1992;132: 619–633. 1385266 10.1093/genetics/132.2.619 PMC1205162

[53] Clement M, Posada DC, Crandall KA. TCS: a computer program to estimate gene genealogies. Mol Ecol. 2000;9: 1657–1659. 10.1046/j.1365-294x.2000.01020.x 11050560

[54] Hart MW, Sunday J. Things fall apart: biological species form unconnected parsimony networks. Biol Lett. 2007;3: 509–512. 10.1098/rsbl.2007.0307 17650475 PMC2391196

[55] Excoffier L, Lischer HEL. Arlequin suite ver 3.5: a new series of programs to perform population genetics analyses under Linux and Windows. Mol Ecol Resour. 2010;10: 564–567. 10.1111/j.1755-0998.2010.02847.x 21565059

[56] Tajima F. Statistical method for testing the neutral mutation hypothesis by DNA polymorphism. Genetics. 1989;123: 585–595. 2513255 10.1093/genetics/123.3.585 PMC1203831

[57] Fu XY. Statistical tests of neutrality of mutations against population growth, hitchhiking and background selection. Genetics. 1997;147: 915–925. 9335623 10.1093/genetics/147.2.915 PMC1208208

[58] Swarts ND, Sinclair EA, Krauss SL, Dixon KW. Genetic diversity in fragmented populations of the critically endangered spider orchid *Caladenia huegelii*: implications for conservation. Conserv Genet. 2009;10: 1199–1208.

[59] Boys J, Cherry M, Dayanandan S. Microsatellite analysis reveals genetically distinct populations of red pine (*Pinus resinosa*, Pinaceae). Am J Bot. 2005;92: 833–841. 10.3732/ajb.92.5.833 21652464

[60] Lee GA, Kwon SJ, Park YJ, Lee MC, Kim HH, Lee JS, et al Cross-amplification of SSR markers developed from *Allium sativum* to other Allium species. Sci Hortic (Amsterdam). 2011;128: 401–407.

[61] Witherup C, Ragone D, Wiesner-Hanks T, Irish B, Scheffler B, Simpson S, et al Development of microsatellite loci in *Artocarpus altilis* (Moraceae) and cross-amplification in congeneric species. Appl Plant Sci. 2013;1: 1200423. 10.3732/apps.1200423 PMC4103128 25202565

[62] Cheng YC, Zhang DJ, Lu ZY, Ye XS, Wang JG, Sun P, et al Isolation and characterization of microsatellite loci for *Prunus mongolica* (Rosaceae). Appl Plant Sci. 2018;6: e01158 10.1002/aps3.1158 30131900 PMC6025809

[63] George S, Sharma J, Yadon VL. Genetic diversity of the endangered and narrow endemic *Piperia yadonii* (Orchidaceae) assessed with ISSR polymorphisms. Am J Bot. 2009;96: 2022–2030. 10.3732/ajb.0800368 21622322

[64] Forrest A, Escudero M, Heuertz M, Wilson Y, Cano E, Vargas P. Testing the hypothesis of low genetic diversity and population structure in narrow endemic species: the endangered *Antirrhinum charidemi* (Plantaginaceae). Bot J Linn Soc. 2017;183: 260–270.

[65] Zhao Y, Pan B, Zhang M. Phylogeography and conservation genetics of the endangered *Tugarinovia mongolica* (Asteraceae) from Inner Mongolia, Northwest China. PLoS ONE. 2019;14: e0211698. 30730930 10.1371/journal.pone.0211696 PMC6366884

[66] Chen YY, Bao ZX, Qu Y, Li W, Li ZZ. Genetic diversity and population structure of the medicinal orchid *Gastrodia elata* revealed by microsatellite analysis. Biochem Syst Ecol. 2014;54: 182–189.

[67] Hamrick JL, Godt MJW. Allozyme diversity in plant species In: Brown AHD, Clegg MT, Kahler AL, Weir BS, editors. Plant population genetics, breeding and genetic resources. Massachusetts: Sinauer Associates; 1989 pp. 43–63.

[68] Cole CT. Genetic variation in rare and common plants. Annu Rev Ecol Evol Syst. 2003;34: 213–237.

[69] Nybom H. Comparison of different nuclear DNA markers for estimating intraspecific genetic diversity in plants. Mol Ecol. 2004;13: 1143–1155. 10.1111/j.1365-294X.2004.02141.x 15078452

[70] Gibson JP, Rice SA, Stucke CM. Comparison of population genetic diversity between a rare, narrowly distributed species and a common, widespread species of *Alnus* (Betulaceae). Am J Bot. 2008;95: 588–596. 10.3732/ajb.2007316 21632385

[71] Loveless MD, Hamrick JL. Ecological determinants of genetic structure of plant populations. Annu Rev Ecol Syst. 1984;15: 65–95.

[72] Hamrick JL, Godt MJ. Effects of life history traits on genetic diversity in plant species. Philos Trans R Soc Lond Ser B Biol Sci. 1996;351: 1291–1298.

[73] Dong YH, Chen JM, Gituru RW, Wang QF. Gene flow in populations of the endangered aquatic fern *Ceratopteris pteridoides* in China as revealed by ISSR markers. Aquat Bot. 2007;87: 69–74.

[74] Gustafsson S, Sjögren-Gulve P. Genetic diversity in the rare orchid *Gymnadenia odoratissima* and a comparison with the more common congener, *G*. *conopsea*. Conserv Genet. 2002;3: 225–234.

[75] Tremetsberger K, Stuessy TF, Samuel RM, Baeza CM, Fay MF. Genetics of colonization in *Hypochaeris tenuifolia* (Asteraceae, Lactuceae) on Volcán Lonquimay, Chile. Mol Ecol. 2003;12: 2649–2659. 10.1046/j.1365-294x.2003.01956.x 12969468

[76] Sharma M, Fomda BA, Mazta S, Sehgal R, Singh BB, Malla N. Genetic diversity and population genetic structure analysis of *Echinococcus granulosus* sensu stricto complex based on mitochondrial DNA signature. PLoS One. 2013;8: e82904 10.1371/journal.pone.0082904 24349394 PMC3857302

[77] Charlesworth D, Charlesworth B. Quantitative genetics in plants: the effect of the breeding system on genetic variability. Evolution. 1995;49: 911–920. 10.1111/j.1558-5646.1995.tb02326.x 28564864

[78] Wolf AT, Harrison SP, Hamrick JL. Influence of habitat patchiness on genetic diversity and spatial structure of a serpentine endemic plant. Conserv Biol. 2000;14: 454–463.

[79] Holderegger R, Stehlik I, Lewis Smith RI, Abbott RJ. Populations of Antarctic hairgrass (*Deschampsia antarctica*) show low genetic diversity. Arct Antarct Alp Res. 2003;35: 214–217.

[80] Ne’eman G, Shavit O, Shaltiel L, Shmida A. Foraging by male and female solitary bees with implications for pollination. J Insect Behav. 2006;19: 383–401.

[81] Janzen DH. Euglossine bees as long-distance pollinators of tropical plants. Science. 1971;171: 203–205. 10.1126/science.171.3967.203 17751330

[82] Qian H, Ricklefs RE. Large-scale processes and the Asian bias in species diversity of temperate plants. Nature. 2000;407: 180–182. 10.1038/35025052 11001054

[83] Harrison SP, Yu G, Takahara H, Prentice IC. Diversity of temperate plants in East Asia. Nature. 2001;413: 129–130. 10.1038/35093166 11557970

