# Supplementary material for: Genetic diversity and population structure of the endangered orchid Pelatantheria scolopendrifolia (Orchidaceae) in Korea
Source: PLoS One. 2020 Aug 13;15(8):e0237546. doi: 10.1371/journal.pone.0237546 (PMC7425873; doi:10.1371/journal.pone.0237546)
Supplement: S1 Table — + and − indicate significant and non-significant level at p < 0.05. (DOCX) [file pone.0237546.s001.docx]

***Supplementary Material***

**Genetic diversity and population structure of the endangered orchid *Pelatantheria scolopendrifolia* (Orchidaceae) in Korea**

**Seon A. Yun^1^, Hyun-Deok Son^2^, Hyoung-Tak Im^3^, Seung-Chul Kim^1*^**

**Correspondence: Seung-Chul Kim:** [**sonchus96@skku.edu**](mailto:sonchus96@skku.edu) **or sonchus2009@gmail.com**

**Supplementary Tables**

**Supplementary Table 1. Linkage disequilibrium over all loci and populations based on microsatellite data.** + and – indicate significant and non-significant level at *p* < 0.05.

|  | PS2 | PS6 | PS24 | PS25 | PS29 | PS31 | PS32 | PS36 | PS37 | PS40 | PS43 | PS44 | PS47 | PS51 | PS52 | CW3164 | CW3189 |
| --- | --- | --- | --- | --- | --- | --- | --- | --- | --- | --- | --- | --- | --- | --- | --- | --- | --- |
| PS6 | + |  |  |  |  |  |  |  |  |  |  |  |  |  |  |  |  |
| PS24 | + | + |  |  |  |  |  |  |  |  |  |  |  |  |  |  |  |
| PS25 | - | + | + |  |  |  |  |  |  |  |  |  |  |  |  |  |  |
| PS29 | - | + | + | - |  |  |  |  |  |  |  |  |  |  |  |  |  |
| PS31 | + | + | + | + | + |  |  |  |  |  |  |  |  |  |  |  |  |
| PS32 | - | + | + | + | - | + |  |  |  |  |  |  |  |  |  |  |  |
| PS36 | + | + | + | + | - | + | + |  |  |  |  |  |  |  |  |  |  |
| PS37 | - | + | + | + | + | + | + | + |  |  |  |  |  |  |  |  |  |
| PS40 | + | + | + | - | + | + | - | - | + |  |  |  |  |  |  |  |  |
| PS43 | - | + | + | + | - | + | + | + | + | - |  |  |  |  |  |  |  |
| PS44 | - | + | + | + | + | + | + | + | + | - | - |  |  |  |  |  |  |
| PS47 | - | - | - | + | + | + | + | + | - | - | + | - |  |  |  |  |  |
| PS51 | + | + | + | - | + | + | + | + | + | + | + | + | + |  |  |  |  |
| PS52 | - | - | + | + | - | + | + | - | - | - | - | - | + | - |  |  |  |
| CW3164 | + | + | + | + | + | + | + | + | - | + | + | + | + | + | + |  |  |
| CW3189 | + | + | + | + | + | + | + | + | + | + | + | + | + | + | + | + |  |
| CW3248 | + | + | + | + | + | + | + | + | + | + | + | + | + | + | + | + | + |
