# Supplementary material for: Genetic diversity and population structure of the endangered orchid Pelatantheria scolopendrifolia (Orchidaceae) in Korea
Source: PLoS One. 2020 Aug 13;15(8):e0237546. doi: 10.1371/journal.pone.0237546 (PMC7425873; doi:10.1371/journal.pone.0237546)
Supplement: S2 Table — Probability values are all under the 0.005. (DOCX) [file pone.0237546.s002.docx]

**Genetic diversity and population structure of the endangered orchid *Pelatantheria scolopendrifolia* (Orchidaceae) in Korea**

**Seon A. Yun^1^, Hyun-Deok Son^2^, Hyoung-Tak Im^3^, Seung-Chul Kim^1*^**

**Correspondence: Seung-Chul Kim:** [**sonchus96@skku.edu**](mailto:sonchus96@skku.edu) **or sonchus2009@gmail.com**

**Supplementary Tables**

**S2 Table. *F_ST_* values among pairs of populations.** Probability values are all under the 0.005.

|  | NJ | MP-1 | MP-2 | HN-1 | HN-2 | JD-1 | JD-2 | GM | WD | JPN-1 |
| --- | --- | --- | --- | --- | --- | --- | --- | --- | --- | --- |
| MP-1 | 0.209 |  |  |  |  |  |  |  |  |  |
| MP-2 | 0.105 | 0.216 |  |  |  |  |  |  |  |  |
| HN-1 | 0.13 | 0.253 | 0.163 |  |  |  |  |  |  |  |
| HN-2 | 0.149 | 0.341 | 0.226 | 0.229 |  |  |  |  |  |  |
| JD-1 | 0.095 | 0.165 | 0.105 | 0.12 | 0.161 |  |  |  |  |  |
| JD-2 | 0.094 | 0.272 | 0.141 | 0.185 | 0.174 | 0.073 |  |  |  |  |
| GM | 0.184 | 0.354 | 0.199 | 0.241 | 0.21 | 0.163 | 0.117 |  |  |  |
| WD | 0.135 | 0.345 | 0.189 | 0.223 | 0.246 | 0.179 | 0.172 | 0.271 |  |  |
| JPN-1 | 0.367 | 0.541 | 0.366 | 0.416 | 0.318 | 0.323 | 0.333 | 0.266 | 0.424 |  |
| JPN-2 | 0.404 | 0.57 | 0.401 | 0.478 | 0.398 | 0.373 | 0.38 | 0.358 | 0.506 | 0.491 |
