# Supplementary material for: Genetic diversity and population structure of the endangered orchid Pelatantheria scolopendrifolia (Orchidaceae) in Korea
Source: PLoS One. 2020 Aug 13;15(8):e0237546. doi: 10.1371/journal.pone.0237546 (PMC7425873; doi:10.1371/journal.pone.0237546)
Supplement: S3 Table — Question mark means missing data. * indicates the sequencing modification. T and A on 633 site were originally TCTTAATAT and 9 bp deletion, respectively; On 665 site, 1 bp deletion character was coded as A; T and C on 775 site stand for TAAGG and CCTTA, respectively. (DOCX) [file pone.0237546.s003.docx]

***Supplementary Material***

**Genetic diversity and population structure of the endangered orchid *Pelatantheria scolopendrifolia* (Orchidaceae) in Korea**

**Seon A. Yun^1^, Hyun-Deok Son^2^, Hyoung-Tak Im^3^, Seung-Chul Kim^1*^**

**Correspondence: Seung-Chul Kim:** [**sonchus96@skku.edu**](mailto:sonchus96@skku.edu) **or sonchus2009@gmail.com**

**Supplementary Tables**

**Supplementary Table 3. Variable sites found *in Pelatantheria scolopendrifolia* identifying 20 haplotypes.** Question mark means missing data.

| Haplotype | *acc*D*-psb*I | | | | | | | | *pet*A-*psb*J | | | | | *rps*12-*rpl*20 | | *pet*L-*psb*E | | | |
| --- | --- | --- | --- | --- | --- | --- | --- | --- | --- | --- | --- | --- | --- | --- | --- | --- | --- | --- | --- |
|  | 420 | 600 | 603 | 633* | 649 | 665* | 724 | 725 | 775* | 1107 | 1302 | 1328 | 1394 | 1554 | 2024 | 2167 | 2170 | 2173 |  |
| A | A | T | T | A | T | G | C | G | C | G | G | T | C | G | A | C | G | T |  |
| B | A | T | T | A | T | G | C | G | C | G | G | T | C | G | G | C | G | T |  |
| C | A | T | T | A | T | G | C | G | C | G | G | T | C | A | G | C | G | T |  |
| D | A | T | T | A | T | A | C | G | C | G | G | T | C | G | A | C | G | T |  |
| E | A | G | T | A | T | G | C | G | C | G | G | T | C | G | A | C | G | T |  |
| F | C | ? | ? | ? | ? | ? | ? | ? | C | G | G | T | C | G | A | C | G | T |  |
| G | C | G | T | A | T | G | A | G | C | G | G | T | C | A | A | A | C | G |  |
| H | C | G | T | A | T | G | A | G | T | G | G | T | C | A | G | A | C | G |  |
| I | C | C | T | A | T | G | A | G | T | G | G | T | C | A | G | A | C | G |  |
| J | C | T | T | A | T | G | A | G | T | G | G | T | C | A | G | A | C | G |  |
| K | C | T | T | A | T | G | A | C | T | G | G | T | C | A | A | A | C | G |  |
| L | C | T | T | A | T | G | A | G | T | G | T | T | C | A | G | A | C | G |  |
| M | C | T | T | A | A | G | A | G | T | G | G | T | C | A | G | A | C | G |  |
| N | C | T | T | A | T | G | A | G | T | G | G | T | C | A | G | A | C | G |  |
| O | C | T | T | T | T | G | A | G | T | T | G | T | A | A | A | A | C | G |  |
| P | C | T | T | T | T | A | A | G | T | T | G | T | A | A | A | A | C | G |  |
| Q | C | G | T | T | T | A | A | G | T | T | G | T | A | A | A | A | C | G |  |
| R | C | G | T | T | T | G | A | G | T | T | G | T | A | A | A | A | C | G |  |
| S | C | G | T | T | T | G | A | G | T | T | G | G | ? | A | A | A | C | G |  |
| T | C | G | A | T | T | G | A | G | T | T | G | T | A | A | A | A | C | G |  |

Question mark means missing data. * indicates the sequencing modification. T and A on 633 site were originally TCTTAATAT and 9 bp deletion, respectively; On 665 site, 1 bp deletion character was coded as A; T and C on 775 site stand for TAAGG and CCTTA, respectively.
