# Supplementary material for: Genetic diversity and population structure of the endangered orchid Pelatantheria scolopendrifolia (Orchidaceae) in Korea
Source: PLoS One. 2020 Aug 13;15(8):e0237546. doi: 10.1371/journal.pone.0237546 (PMC7425873; doi:10.1371/journal.pone.0237546)
Supplement: S4 Table — (a) Result based on microsatellite data. (b) Result based on chloroplast DNA data. For this analysis, three population types: coastal, inland, and island, were set as region. Regions: inland (NJ, HN-1), coastal (MP-1, MP-2, and HN-2), and island (JD-1, JD-2, WD, GM, JPN-1, and JPN-2). (DOCX) [file pone.0237546.s004.docx]

**Genetic diversity and population structure of the endangered orchid *Pelatantheria scolopendrifolia* (Orchidaceae) in Korea**

**Seon A. Yun^1^, Hyun-Deok Son^2^, Hyoung-Tak Im^3^, Seung-Chul Kim^1*^**

**Correspondence: Seung-Chul Kim:** [**sonchus96@skku.edu**](mailto:sonchus96@skku.edu) **or sonchus2009@gmail.com**

**Supplementary Tables**

**S4 Table. Analyses of molecular variance using microsatellite and chloroplast DNA data.** (a) Result based on microsatellite data. (b) Result based on chloroplast DNA data. For this analysis, three population types: coastal, inland, and island, were set as region. Regions: inland (NJ, HN-1), coastal (MP-1, MP-2, and HN-2), and island (JD-1, JD-2, WD, GM, JPN-1, and JPN-2).

(a)

| **Source** | ***d. f.*** | **SS** | **Variation (%)** |
| --- | --- | --- | --- |
| Among Regions | 2 | 189.726 | 0% |
| Among Populations | 8 | 805.786 | 55% |
| Within Populations | 171 | 957.383 | 45% |
| Total | 181 | 1952.896 | 100% |

* *d. f.* degree of freedom, SS sum of squares

(b)

| **Source of variation** | ***d. f.*** | **SS** | **VC** | **PV (%)** | **Fixation indices** | ***p* value** |
| --- | --- | --- | --- | --- | --- | --- |
| Among regions | 2 | 107.576 | 0.22971 | 7.41 | F_CT_= 0.07410 | 0.29814±0.01534 |
| Among populations within region | 8 | 220.943 | 1.95479 | 63.06 | F_SC_=0.68109 | 0.00000±0.00000 |
| Within populations | 171 | 156.514 | 0.91529 | 29.53 | F_ST_= 0.70473 | 0.00000±0.00000 |
| Total | 181 | 485.033 | 3.09978 |  |  |  |

* *d. f*. degree of freedom, SS sum of squares, VC variance components, PV percentage of variation.
