# Supplementary figures and images for: Genetic diversity and population structure of the endangered orchid Pelatantheria scolopendrifolia (Orchidaceae) in Korea
Source: PLoS One. 2020 Aug 13;15(8):e0237546. doi: 10.1371/journal.pone.0237546 (PMC7425873; doi:10.1371/journal.pone.0237546)

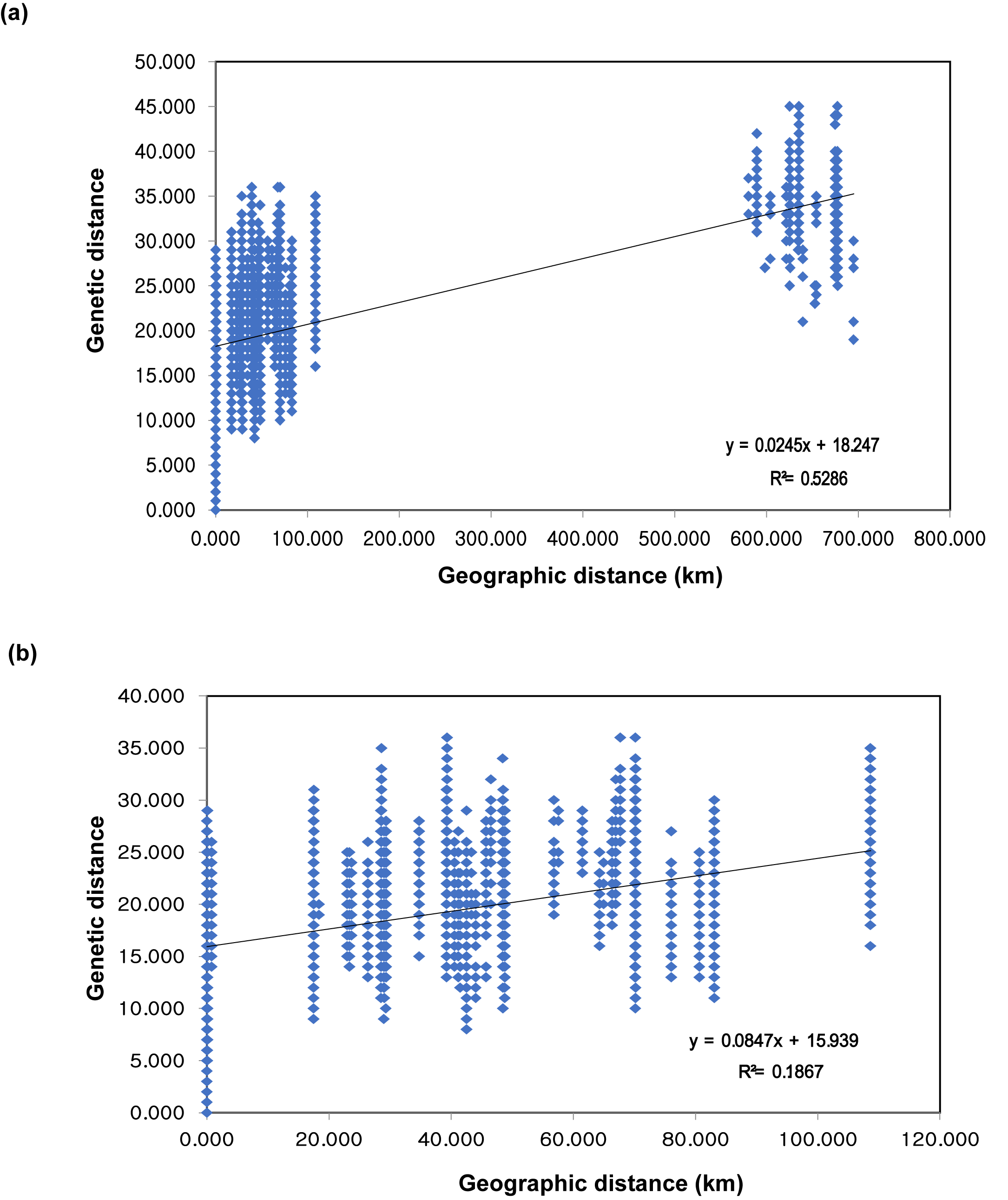

Supplement: S1 Fig — (a) all populations. (b) Korean populations. The results of the Mantel test indicate the positive correlation between genetic and geographic distances was significant. Note: P = 0.001 for all correlations. (TIF) [file pone.0237546.s005.tif]
